# Heartbeat, embryo communication and hatching synchrony in snake eggs

**DOI:** 10.1038/srep23519

**Published:** 2016-03-18

**Authors:** Fabien Aubret, Gaëlle Blanvillain, Florent Bignon, Philippe J. R. Kok

**Affiliations:** 1 Station d’Ecologie Théorique et Expérimentale, CNRS, UMR 5321, 09200 Moulis, France; 2 Amphibian Evolution Lab, Biology Department, Vrije Universiteit Brussel, 2 Pleinlaan, B-1050 Brussels, Belgium

## Abstract

Communication is central to life at all levels of complexity, from cells to organs, through to organisms and communities. Turtle eggs were recently shown to communicate with each other in order to synchronise their development and generate beneficial hatching synchrony. Yet the mechanism underlying embryo to embryo communication remains unknown. Here we show that within a clutch, developing snake embryos use heart beats emanating from neighbouring eggs as a clue for their metabolic level, in order to synchronise development and ultimately hatching. Eggs of the water snake *Natrix maura* increased heart rates and hatched earlier than control eggs in response to being incubated in physical contact with more advanced eggs. The former produced shorter and slower swimming young than their control siblings. Our results suggest potential fitness consequences of embryo to embryo communication and describe a novel driver for the evolution of egg-clustering behaviour in animals.

Cells, organs and whole organisms communicate both within and amongst species^1^. Animals may acquire or pass on information using visual, acoustic, chemical or mechanical channels. Obvious examples such as the human spoken language^2^, alarm calls in meerkats^3^ or aposematic signals in frogs^4^ have evolved as potent information conveyers and survival enhancers. Yet, the exchange of information may be more subtle, as is the case for informed dispersal in the common lizard^5^ or embryo to embryo communication within a clutch in both avian and non**-**avian reptiles^6^,^7^,^8^,^9^,^10^. In the latter, cues such as sound production, egg vibration, an increase in heart rates, odours, or carbon dioxide levels within the nest were proposed as potential communication avenues amongst embryos^6^,^7^,^8^,^9^,^10^. We thought heart rate levels to be particularly promising given that (i) most reptile eggs and indeed most eggs in the animal kingdom are either laid in contact with each other or in clusters where eggs are strongly adhered to each other^11^,^12^; (ii) shockwaves travel well in liquids^13^; (iii) egg shells vibrate (the heart rate monitor used in this study is designed to detect minute distortions caused by embryonic heart beats^14^); and (iv) water snake embryos were shown to adjust heart rates (an estimate of development rate) and generate intra**-**clutch hatching synchrony^15^.

Synchronised hatching is widespread amongst organisms, including reptiles^8^,^16^, and is thought to enhance offspring survival by diluting an individual’s risk of predation or by simply swamping predators upon emergence^17^,^18^. Turtles typically lay eggs in a shallow underground nest that is characterised by a top to bottom temperature gradient where top eggs experience incubation temperatures up to 6 °C warmer than bottom eggs^19^. Yet, despite this gradient and the fact that embryo development is strongly linked to incubation temperature^20^, all eggs hatch in synchrony^7^. Experimental evidence^7^,^8^ suggests that bottom eggs (i.e. low incubation temperature) promote synchronous hatching either by (i) accelerating development (coined the “catch up hypothesis”) or (ii) hatching prematurely with potential detrimental effects (i.e. a drop in locomotor performance^7^,^8^) that are outweighed in the long run by the benefits of synchronous hatching^17^,^18^.

Snakes do not actively bury their eggs but use natural cavities, where intra- as well as inter-specific communal nesting is common^21^,^22^,^23^. Hence, while most eggs may experience similar incubation temperatures (i.e. in the absence of top to bottom temperature gradient), it is likely that new clutches will be laid close to or in direct contact with older clutches laid hours or days earlier by other snakes. In fact, it is known that female squamates will actively lay their eggs next to others rather than choose an isolated but identical location^24^. We sought to recreate and take advantage of this natural experiment to investigate the potential influence older embryos might have on the development of younger eggs (i.e. generate faster development or premature hatching) and test the possibility that younger eggs respond to the perceived heart rates of older eggs as a proxy for metabolic levels (heart rates steadily increase throughout the incubation period in water snakes^15^; Fig. 1). For this purpose 6 freshly laid snake clutches (77 eggs) were split into 12 half clutches of which 6 were incubated in close contact with slightly older eggs and the remaining 6 were used as controls. We recorded heart rates for each egg throughout the incubation period, hatching success and hatching phenotypes of the newly hatched snakes.

## Results

### Experimental design

**Figure 1:**
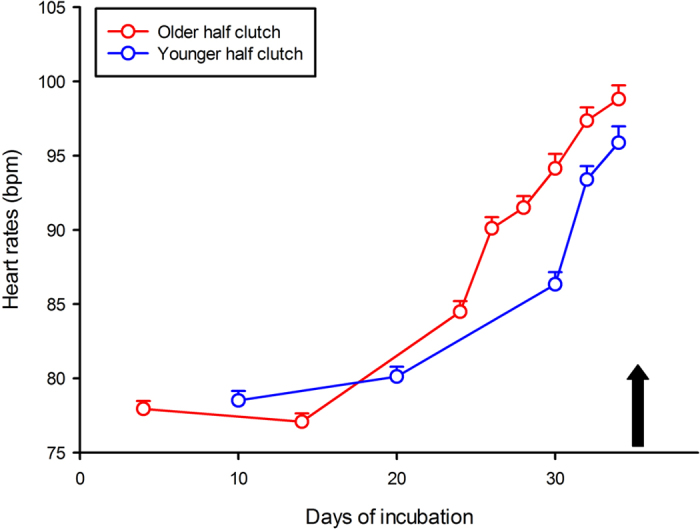
Six freshly laid clutches of the water snake *Natrix maura* were split into 12 half clutches: 6 half clutches were incubated in close contact with slightly older eggs (mixed) and the remaining 6 half clutches were used as controls. Heart rates (Y axis) were recorded for each egg throughout the incubation period (X axis). Heart rates naturally increase during the incubation, therefore eggs mixed with older eggs were consistently exposed, from incubation day 17 onwards, and until older eggs hatched (black arrow) to higher heart rates (on average 89.81 ± 3.92 bpm) than they would have experienced if not mixed (i.e. control eggs averaged 82.52 ± 3.39 bpm). Means ± SE are plotted.

**Figure 2:**
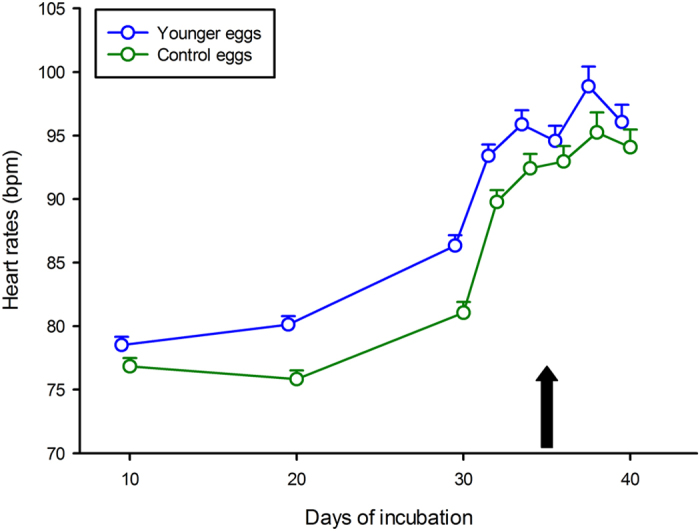
Heart rates (Y axis) for the entire incubation period (X axis) for younger embryos (6 half clutches mixed with older clutches) and for control siblings (the remaining 6 half clutches). Younger embryo heart rates averaged 92.73 ± 5.23 bpm throughout the incubation *versus* 84.76 ± 7.68 bpm in (control) sibling embryos. The black arrow indicates the hatching time of older eggs, after which younger embryos were by themselves. Means ± SE are plotted.

Eggs (hereafter called “younger” eggs) mixed with older eggs (i.e. born 5.8 ± 1.7 days earlier on average) were consistently exposed, from incubation day 17 onwards, and until older eggs hatched, to higher heart rates (on average 92.73 ± 5.23 bpm; Fig. 1) than they would have experienced if not mixed (i.e. control eggs averaged 84.76 ± 7.68 bpm over the same timeframe**-**F_1, 72_ = 85.51, P < 0.0001 – see Fig. 2).

### Younger eggs versus control eggs

A repeated measure ANOVA with “clutch of origin” and “treatment” as factors and 8 successive heart rates as the repeated measure through time yielded a significant result (Wilks lambda = 0.53, F_8, 46_ = 5.08, P < 0.00015). There was no significant interaction between the two factors (F_7, 371_ = 0.81, P = 0.58) but a significant effect of treatment (F_1, 53_ = 21.36, P < 0.0001): younger egg heart rates averaged 90.47 ± 2.69 bpm throughout the incubation *versus* 87.27 ± 2.88 bpm in (control) sibling eggs.

Due to increased mean heart rates, younger eggs developed faster and hatched half a day earlier than control eggs (incubation duration was 43.50 ± 0.85 days in the former *versus* 43.98 ± 0.92 days in the latter; F_1, 45_ = 4.14, P = 0.048). Faster development altered hatchling body size: snakes from younger clutches were shorter in snout-vent length (12.23 ± 0.66 cm *versus* 12.73 ± 0.63 cm respectively; F_1, 46_ = 8.62, P < 0.0052) and in better condition than sibling snakes from control eggs (0.0077 ± 0.052 versus −0.022 ± 0.050 respectively; F_1, 46_ = 4.73, P < 0.035). Body mass was unaffected by the treatment (F_1, 46_ = 0.39, P = 0.54). Finally, snakes from younger eggs were slower swimmers than their siblings both in absolute terms (36.17 ± 8.77 cm.s^−1^ vs 43.73 ± 7.43 cm.s^−1^ respectively; F_1, 38_ = 10.28, P < 0.0027) and relative to body length (F_1, 38_ = 12.16, P < 0.0013).

## Discussion

This simple experiment demonstrated that within a clutch, snake embryos are able to alter heart rates (i.e. metabolic levels) in response to the perceived heart beats of surrounding embryos. Eggs incubated in contact with older eggs, that is, exposed to higher heart rates, responded by accelerating development and hatched half a day earlier than control eggs. This result (i) highlights a mechanism (heart beats) by which intra-clutch hatching synchrony may be achieved in snakes^13^ and (ii) is in accordance with the catch-up hypothesis^7^ where turtle eggs from the (relatively colder) bottom of the nest accelerated their development in an attempt to synchronously hatch with eggs from the (relatively warmer) top of the nest. Faster development and early hatching altered yolk assimilation (young snakes were born shorter, but with higher fat storage) and came at a cost: swimming performance was reduced in early hatchers compared to sibling control snakes.

Egg-clustering and communal nesting behaviour in reptiles and indeed in most egg laying species, were traditionally assumed to have evolved primarily as anti-predatory tactics (from extinct dinosaurs^25^ through to insects^26^, spiders^27^, cephalopods^28^, fish^29^ to amphibians^30^). Recent studies also reported direct benefits of egg clustering and communal nesting to offspring phenotypes through hydric modifications of incubation conditions^24^ or through securing and maintaining egg position^12^. Our results add a novel dimension to the evolution of egg-clustering and communal nesting in the animal kingdom in the form of embryo to embryo communication: the kind of information exchanged (metabolic rate, stress, sex ratio, relatedness, clutch size etc.) may convey important information about the quality of the natal environment as well as an estimate of forthcoming competition amongst mates, conspecifics and heterospecifics. As such, long term effects of embryo to embryo communication (personality, dispersal) are a new, yet fascinating field to be researched at a traditionally overlooked life-stage.

## Material and Methods

### Experimental design

Gravid females were captured along the banks of the Lez River in South-West Ariège, France, in June and July 2014. A total of 77 eggs were obtained from 6 clutches between 14/07/2014 and 07/08/2014 (mean clutch size = 12.8 ± 5.9 eggs). Eggs were collected within 12 hours post laying. Eggs were individualised by gently rupturing the natural contact areas. This procedure greatly reduced the possibility of chemical communication amongst eggs; although there is no evidence that eggs may exchange chemical substances such as steroid hormones (Spencer & McGlashan, pers. com.). Eggs were individually marked for identification purposes with a pencil using a letter (coding for clutch of origin) and a number (egg number within each clutch). Egg mass influences both embryo metabolism and hatching phenotype^15^, so eggs were allocated to two treatments using a split clutch design: eggs were numbered within each clutch from heaviest to lightest and evenly reunited into two half-clutches. This ensured consistency in egg mass across treatments (two**-**factors Nested ANOVA with female nested into half-clutch: F_1, 65_ = 0.30, P = 0.59). Six of the twelve half clutches (N = 6.67 ± 3.01 eggs on average) were merged and mixed with older eggs (N = 6.50 ± 2.26 eggs on average) obtained from another 6 clutches laid a few days earlier (between 09/07/2014 and 03/08/2014) to generate 6 experimental mixed clutches (average difference in age between eggs was 5.8 ± 1.7 days (span: 4 to 9 days). The remaining half clutches (N = 6.2 ± 2.9 eggs on average) were incubated along with the 6 experimental mixed clutches in an Aqualytic^®^ incubation chamber set at a constant 28 °C. Eggs were placed in a plastic container (one clutch per container; 20 cm × 15 cm × 5 cm) on a 2 cm layer of vermiculite.

### Egg and hatchling measurements

Eggs were measured in mass to the nearest 0.01 g using a digital scale within 12 hours of oviposition. Eggs were individually placed into small jewellery bags (5 × 4 cm, made of fine mesh material) a few days prior to hatching. This ensured juvenile snakes could be matched to their egg shell when multiple births occurred at the same time, while maintaining physical contact amongst eggs. Hatchlings were measured in body mass ( ±0.01 g) and snout**-**vent length ( ±0.1 mm) within 12 hours of emergence. Siblings were housed together in plastic boxes (15 cm × 10 cm × 5 cm) with a water dish, shelter and paper towel as substrate. A Body Condition Index (BCI) was calculated for each snake, using the residual values of the linear least**-**squares regression of Log (birth body mass) against Log (birth snout**-**vent length). All tests were performed on all hatchlings.

### Heart rate measurements

We measured embryo heart rates using the Buddy^®^ digital egg monitor (MK2, Avitronics) under the standardised protocol described for eggs^12^,^15^. The Buddy^®^ system works by shining an infrared beam onto the surface of the egg, detecting minute distortions caused by embryonic heart beats. The Buddy^®^ monitor was left inside the incubator at all times to prevent temperature variation during heart rate readings. Each egg was gently placed onto the sensor pad for heart rate reading (a stable reading was obtained after approximately 30 seconds) and then returned to its clutch. Embryo heart rates were measured at incubation day 10, 20 and 30, and then every two days until hatching.

### Swimming performance

A high definition wide angle digital camera (25 fps) was fitted above a linear 300 cm × 40 cm × 50 cm swimming track and used to record trials (recording section of 120 cm). The tank was filled with 10 cm of water adjusted to 25 °C using a reverse-cycle water chiller (TECO^®^ TC15). The video was then edited on a computer and swimming speed calculated over the first five lengths of the track swum by each snake. The fastest performance was retained for each of the five lengths swum, and a fastest overall performance for the entire swimming test (the first length was usually the fastest).

All experimental protocols were approved by the Préfecture de l’Ariège, which provided capture, breeding, experimentation, release and ethics permits (Arrété #2012**-**11). All experiments were carried out in accordance with the approved guidelines. All females were returned to their exact site of capture within two weeks of egg**-**laying. Once tests were completed, young snakes were given their first meal (small dead minnows ranging from 0.5 g to 1 g; supplied by Armorvif ^®^) prior to being released at the maternal capture site.

### Data analysis

Assumptions for normality of the data and equality of variances were tested on all variables (Lilliefors and Levene’s tests). Means ± standard deviations are given unless otherwise stated.

## Additional Information

**How to cite this article**: Aubret, F. *et al*. Heartbeat, embryo communication and hatching synchrony in snake eggs. *Sci. Rep.* **6**, 23519; doi: 10.1038/srep23519 (2016).

## References

[b1] Bradbury J. W. & Vehrencamp S. L. Principles of animal communication (Sinauer Associates, 1998).

[b2] Larson R. K., Déprez V. & Yamakido H. The evolution of human language (Cambridge University Press, 2010).

[b3] Manser M. B. The acoustic structure of suricates’ alarm calls varies with predator type and the level of response urgency. Proc. Roy. Soc. Lond. B 268(1483), 2315–2324 (2001). 10.1098/rspb.2001.1773 PMC1088882 11703871

[b4] Maan M. E. & Cummings M. E. Poison frog colors are honest signals of toxicity, particularly for bird predators. Am. Nat. 179(1), E1–E14 (2012). 22173468 10.1086/663197

[b5] Clobert J. . Informed dispersal, heterogeneity in animal dispersal syndromes and the dynamics of spatially structured populations. Ecol. lett. 12(3), 197–209 (2009). 19170731 10.1111/j.1461-0248.2008.01267.x

[b6] Schwagmeyer P. L. . Effects of Sibling Contact on Hatch Timing in an Asynchronously Hatching Bird. Anim. Behav. 41, 887–894 (1991).

[b7] Spencer R. J., Thompson M. B. & Banks P. B. Hatch or wait? A dilemma in reptilian incubation. Oikos 93, 401–406 (2001).

[b8] McGlashan J. K., Spencer R. J. & Old J. M. Embryonic communication in the nest: metabolic responses of reptilian embryos to developmental rates of siblings. Proc. Roy. Soc. Lond. B 279(1734), 1709–1715 (2012). 10.1098/rspb.2011.2074 PMC3297455 22130606

[b9] McGlashan J. K., Loudon F. K., Thompson M. B. & Spencer R. J. Hatching behavior of eastern long-necked turtles (*Chelodina longicollis*): The influence of asynchronous environments on embryonic heart rate and phenotype. Comp. Biochem. Physiol. A: Mol Integr. Physiol. 188, 58–64 (2015). 26119599 10.1016/j.cbpa.2015.06.018

[b10] Webster B., Hayes W. & Pike T. W. Avian egg odour encodes information on embryo sex, fertility and development. PloS one 10(1), e0116345 (2015). 25629413 10.1371/journal.pone.0116345 PMC4309571

[b11] Packard G. C. & Packard M. J. The physiological ecology of reptilian eggs and embryos (Smith VII, 1988). 10.1111/j.1469-185x.1977.tb01346.x 319843

[b12] Aubret F., Blanvillain G. & Kok P. J. R. Myth busting? Effects of embryo positioning and egg rolling on hatching success in the water snake *Natrix maura*. Sci. rep. 5, doi: 10.1038./srep123385 (2015). PMC4543940 26294250

[b13] Hachiya H. . Determination of sound speed in biological tissues based on frequency analysis of pulse response. J. Acoust. Soc. Am. 92(3), 1564–1568 (1992). 1401526 10.1121/1.403897

[b14] Du W. G., Radder R. S., Sun B. & Shine R. Determinants of incubation period: do reptilian embryos hatch after a fixed total number of heart beats? J. Exp. Biol. 212, 1302–1306 (2009). 19376951 10.1242/jeb.027425

[b15] Aubret F. Heart rates increase after hatching in two species of natricine snakes. Sci. Rep. 3, doi: 10.1038/srep03384 (2013). PMC3843164 24287712

[b16] Colbert P. L., Spencer R. J. & Janzen F. J. Mechanism and cost of synchronous hatching. Funct. Ecol. 24(1), 112–121 (2010).

[b17] Arnold S. J. & Wassersug R. J. Differential predation on metamorphic anurans by garter snakes (*Thamnophis*): social behavior as a possible defense. Ecology 59, 1014–1022 (1978).

[b18] Dehn M. M. Vigilance for predators: detection and dilution effects. Behav. Ecol. Sociobiol. 26, 337–342 (1990).

[b19] Thompson M. B. Nest temperatures in the pleurodiran turtle, Emydura macquarii. Copeia, 1988, 996–1000 (1988).

[b20] Thompson M. B. Egg physiology and biology (TFH Publications Inc., New Jersey, 1997).

[b21] Madsen T. Movements, home range size and habitat use of radio-tracked grass snakes (*Natrix natrix*) in southern Sweden. Copeia 1984, 707–713 (1984).

[b22] Graves B. M. & Duvall D. Aggregation of squamate reptiles associated with gestation, oviposition, and parturition. Herp. Mono 1995, 102–119 (1995).

[b23] Doody J. S., Freedberg S. & Keogh J. S. Communal egg-laying in reptiles and amphibians: evolutionary patterns and hypotheses. Quart. Rev. Biol. 84(3), 229–252 (2009). 19764282 10.1086/605078

[b24] Radder R. S. & Shine R. Why do female lizards lay their eggs in communal nests? J. Anim. Ecol. 76(5), 881–887 (2007). 17714266 10.1111/j.1365-2656.2007.01279.x

[b25] Varricchio D. J., Jackson F. & Trueman C. N. A nesting trace with eggs for the Cretaceous theropod dinosaur Troodon formosus. J. Vert. Paleontol. 19(1), 91–100 (1999).

[b26] Courtney S. P. The evolution of egg clustering by butterflies and other insects. Am. Nat. 1984, 276–281 (1984).

[b27] Buskirk R. E. Sociality in the Arachnida. Social Insects 2, 281–367 (1981).

[b28] Ansell A. D. . Cephalopod eggs and egg masses. Oceanogr. Mar. Biol. 36, 341–71 (1998).

[b29] Ishimatsu A. & Graham J. B. Roles of environmental cues for embryonic incubation and hatching in mudskippers. Integr. Comp. Biol. 51(1), 38–48 (2011). 21705800 10.1093/icb/icr018

[b30] Duellman W. E. & Trueb L. Biology of amphibians (JHU Press, 1986).

